# Doxycycline induces dysbiosis in female C57BL/6NCrl mice

**DOI:** 10.1186/s13104-017-2960-7

**Published:** 2017-11-29

**Authors:** Felicia D. Duke Boynton, Aaron C. Ericsson, Mayu Uchihashi, Misha L. Dunbar, J. Erby Wilkinson

**Affiliations:** 1 Unit for Laboratory Animal Medicine, University of Michigan Medical School, Ann Arbor, MI USA; 2 Research Animal Resources, University of Minnesota, Minneapolis, MN USA; 3 University of Missouri Metagenomics Research Center, University of Missouri, Columbia, MO USA; 4 Medtronic Innovation Center Japan, Medtronic Japan Co., Ltd. Kawasaki, Kanagawa, Japan; 5 Department of Pathology, University of Michigan Medical School, Ann Arbor, MI USA

**Keywords:** Doxycycline, Tet-on, Tet-off, Microbiome, Microbiota, Mouse, Animal model

## Abstract

**Objective:**

This study aims to demonstrate the effect of oral doxycycline on fecal microbiota of mice. Doxycycline is a common effector for control of gene expression using the tet-inducible system in transgenic mice. The effect of oral doxycycline on murine gut microbiota has not been reported. We evaluated the effect of doxycycline treatment by sequencing the V4 hypervariable region of the 16S rRNA gene from fecal samples collected during a 4 week course of treatment at a dose of 2 mg/ml in the drinking water.

**Results:**

The fecal microbiota of treated animals were distinct from control animals; the decreased richness and diversity were characterized primarily by *Bacteroides* sp. enrichment. These effects persisted when the treatment was temporarily discontinued for 1 week. These data suggest that doxycycline treatment can induce significant dysbiosis, and its effects should be considered when used in animal models that are or maybe sensitive to perturbation of the gut microbiota.

**Electronic supplementary material:**

The online version of this article (10.1186/s13104-017-2960-7) contains supplementary material, which is available to authorized users.

## Introduction

Altered gut microbiota (GM) has been associated with a growing list of human conditions (e.g. colorectal cancer [1, 2], inflammatory bowel disease [3, 4], rheumatoid arthritis [5]) and animal models (e.g. type 2 diabetes [6–8], multiple sclerosis [9], anxiety [10], colon cancer [11], arthritis [12], atherosclerosis [13, 14]). Furthermore, the GM plays an important role in many mammalian physiologic processes such as energy balance and metabolism [15], immune function [16–18], angiogenesis [19, 20], and brain development and behavior [21–24]. This suggests that the GM of mouse models should be considered as a variable in animal experiments.

Tetracycline-inducible models have become increasing popular since their creation in the 1990s, though doxycycline’s efficacy has made it the preferred effector over tetracycline [25]. A PubMed search for “mouse” and “doxycycline” yields over 2000 articles describing tet-inducible mouse models of heart failure [26], memory and reversal learning [27], mammary tumors [28], type-1 diabetes mellitus [29], colorectal cancer [30], intestinal inflammation [31] and others. This crude metric demonstrates the pervasiveness of doxycycline use in many disciplines. Oral amoxicillin, metronidazole, vancomycin have already been shown to induce significant and sometimes long-lasting GM perturbations in mice [32, 33] but the effect of oral doxycycline on the GM has not been described.

In light of these findings, the authors believe it is important to determine if and how oral doxycycline affects the GM of mice. Demonstrating and characterizing any effect may enable investigators to control for or eliminate microbiota-induced variability in their experimental design.

We hypothesized that oral doxycycline would alter the composition of the GM (as evidenced by changes in the fecal microbiota [FM]) in female C57BL/6NCrl mice, and that the GM would return to baseline when the drug was temporarily discontinued. The C57BL/6 strain was chosen because it is most commonly used to generate transgenic animals. Females were used to allow for group housing with the intention of enhancing animal welfare. The dose of 2 mg/ml is the most commonly used dose at the authors’ institution, and is the approximate median of published doses which range from 0.2 to 7.5 mg/ml [34, 35]. The collection of fecal pellets allowed for longitudinal collection as opposed to collection of ceca or cecal contents, which are terminal procedures.

## Main text

### Methods

#### Animal models

All studies were performed in accordance with the recommendations put forth in the Guide for the Care and Use of Laboratory Animals and were approved by the University of Michigan Institutional Animal Care and Use Committee. Detailed descriptions of animals and husbandry, welfare assessments and interventions, doxycycline administration and sample collection as recommended by the ARRIVE Guidelines are included in Additional file 1.

#### Experimental design

On day 0, 20 female C57BL/6NCrl mice were divided into experimental (DOX, *n* = 10) and control (*n* = 10) groups. No formal randomization was performed. DOX animals were administered doxycycline in the drinking water at a concentration of 2 mg/ml during weeks 1, 2, and 4; they received distilled water during week 3. Animals in the control group received distilled water for the duration of the study. Feces were collected on days 0, 7, 14, 21, and 28 and stored at − 80 °C until analysis. No other experimental manipulations were performed on the animals. At the end of the 4 week study, animals were transferred to another protocol.

#### DNA extraction, quantification and assessment of purity

DNA extraction was performed as previously described [36] (see Additional file 2 for details). DNA concentrations were determined fluorometrically (Qubit dsDNA BR assay, Life Technologies, Carlsbad, CA) and purity was assessed via 260/280 and 260/230 absorbance ratios, as determined via spectrophotometry (Nanodrop 1000 Spectrophotometer, Thermo Fisher Scientific, Waltham, MA). Samples were stored at − 20 °C until sequencing.

#### Library construction and 16S rRNA sequencing

Library construction and sequencing was performed at the University of Missouri DNA Core facility. DNA concentration of samples was determined fluorometrically and all samples were normalized to 3.51 ng/µL for PCR amplification. Bacterial 16S rRNA amplicons were generated via amplification of the V4 hypervariable region of the 16S rRNA gene using single-indexed universal primers (U515F/806R) flanked by Illumina standard adapter sequences and the following parameters: 98 °C (3:00) + [98 °C (0:15) + 50 °C (0:30) + 72 °C (0:30)] × 25 cycles +72 °C (7:00). Amplicons were then pooled for sequencing using the Illumina MiSeq platform and V2 chemistry with 2 × 250 bp paired-end reads, as previously described [36]. Samples returning greater than 10,000 reads were deemed to have successful amplification. All samples were sequenced on two separate plates and the data was concatenated during the informatics stage. All treatment groups and time-points were split as evenly as possible between plates to account for any minor plate effect during sequencing.

#### Informatics analysis

Assembly, binning, and annotation of DNA sequences were performed at the MU Informatics Research Core Facility. Briefly, contiguous DNA sequences were assembled using FLASH software [37] and culled if found to be short after trimming for a base quality less than 31. Qiime v1.8 [38] software was used to perform de novo and reference-based chimera detection and removal, and remaining contiguous sequences were assigned to operational taxonomic units (OTUs) via de novo OTU clustering and a criterion of 97% nucleotide identity. Taxonomy was assigned to selected OTUs using BLAST [39] against the Greengenes database [40] of 16S rRNA sequences and taxonomy. Principal component analyses were performed using ¼ root-transformed OTU relative abundance data via a non-linear iterative partial least squares (NIPALS) algorithm, using an open access Excel macro available from the Riken Institute (http://prime.psc.riken.jp/Metabolomics_Software/StatisticalAnalysisOnMicrosoftExcel/index.html).

#### Statistical methods

Testing for significant differences between groups and time-points in richness and α-diversity was performed using SigmaPlot 13.0. Briefly, data were tested for normality and equal variance. Once confirmed, main effects of treatment and time-point, and interactions between independent variables, were tested via two-way ANOVA. Main effects of treatment and time-point (and interactions) on β-diversity were tested using non-transformed data via two-way PERMANOVA using Past 3.13 [41]. One-way PERMANOVA was performed post hoc to determine pair-wise differences.

### Results

Sequencing at a mean depth of 75407 sequences per sample, between 4 and 146,082 total unique sequences were detected. Rarefaction analysis indicates the number of OTUs detected per sample was independent of sequencing depth above approximately 20,000 sequences indicating the sequencing depth was adequate to detect rare taxa. Twelve samples, all in the DOX group on days 7 and 14, yielded approximately 10,000 reads or fewer (range = 4–10,048) and were omitted from further analysis. A total of 88 samples were included.

#### Doxycycline treatment decreases richness and diversity

**Fig. 1:**
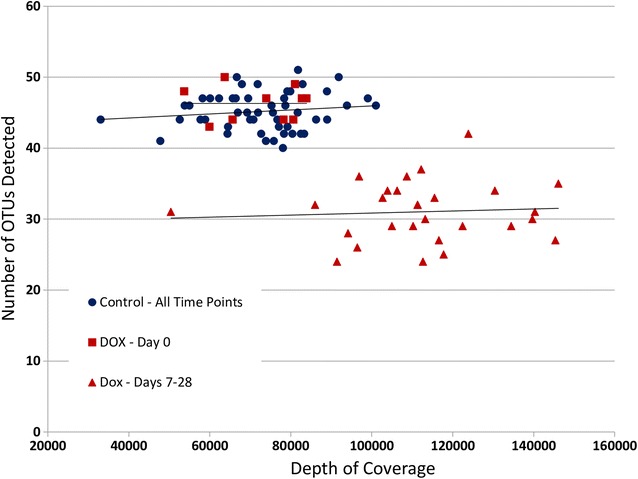
Rarefaction of sequencing data. Rarefaction analysis comparing the number of detected OTUs to the total number of sequences obtained for each individual sample at all time points. Data points are colored to indicate experimental group: Control, blue; DOX, red

Assembly and binning of the raw sequence data from the 88 samples resulted in 23–50 distinct OTUs represented per sample at each time point. The control group contained 40-50 OTUs per sample per time point, with an overall average of 44.2 OTUs per sample. DOX samples collected after treatment range from 23 to 41 OTUs per animal per time point with an overall average of 30 OTUs per sample (Additional file 3). Rarefaction analysis indicated an apparent difference in microbial richness between treatment groups with control animals at all time points clustering above doxycycline-treated animals at all post-treatment time points. DOX samples on day 0 clustered with the control animals (Fig. 1). Comparison of Chao1 and Shannon indices between treatment groups indicates significant decreases in richness and diversity in the DOX animals post-treatment, with significant main effects of time, treatment, and a significant time × treatment interaction (*p* < 0.001, 2-way ANOVA, Additional file 3).

#### Doxycycline treatment alters relative abundance

**Fig. 2:**
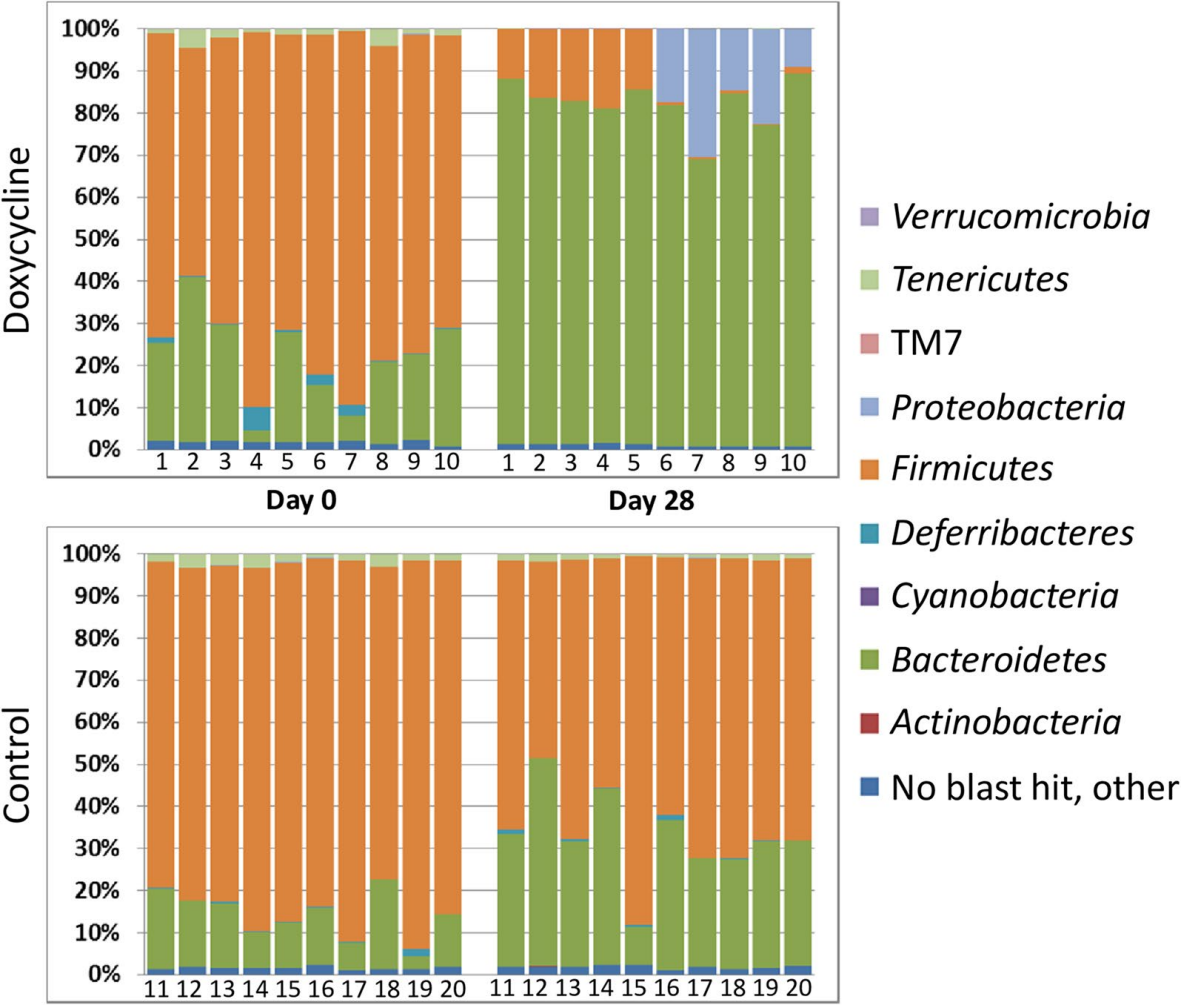
Relative abundance at taxonomic level of phylum. Bar charts showing the bacterial composition of the same control and doxycycline-treated mice on days 0 and 28 annotated to the taxonomic level of phylum. Legend of phyla is shown at right. Each bar represents an individual animal (DOX animals numbered 1–10, control animals numbered 11–20)

The OTUs from all samples were annotated to nine phyla: *Actinobacteria*, *Bacteroidetes*, *Cyanobacteria*, *Deferribacteres*, *Firmicutes*, *Proteobacteria*, *Tenericutes*, TM7, and *Verrucomicrobia* (Fig. 2). As expected, the FM was composed primarily of *Firmicutes* and *Bacteroidetes* in both groups on day 0, the relative abundance of these and other phyla was stable over time in the control animals. Conversely, the relative abundance of these phyla was markedly altered in the DOX samples on day 28, characterized primarily by enrichment of the phylum *Bacteroidetes*. *Firmicutes* were decreased, and samples from DOX cage 2 demonstrate relative enrichment of *Proteobacteria*.

Relative abundance at the OTU level is shown for all time points in Additional file 4. The predominant *Firmicutes* OTU in control animals at all time points, and DOX animals on day 0 was annotated to unclassified (UC) order *Clostridiales*, with an average relative abundance of 37.3% in the control animals at all points and 40.1% in the DOX animals on day 0. At the same time points, most *Bacteroidetes* species were annotated to *Bacteroides* and fewer *Parabacteroides*. Conversely, *Bacteroides* species dominated the DOX samples on days 7–28, accounting for an average of 75.0% of all reads in post-treatment samples. In these same samples, the relative abundance of UC order *Clostridiales* dropped from the day 0 average of 40.1% to 3.4%. In these samples, the marked decrease in UC order *Clostridiales* was accompanied by an increase in UC family *Enterobacteriaceae*.

#### Doxycycline-treated animals have distinct populations from control mice

**Fig. 3:**
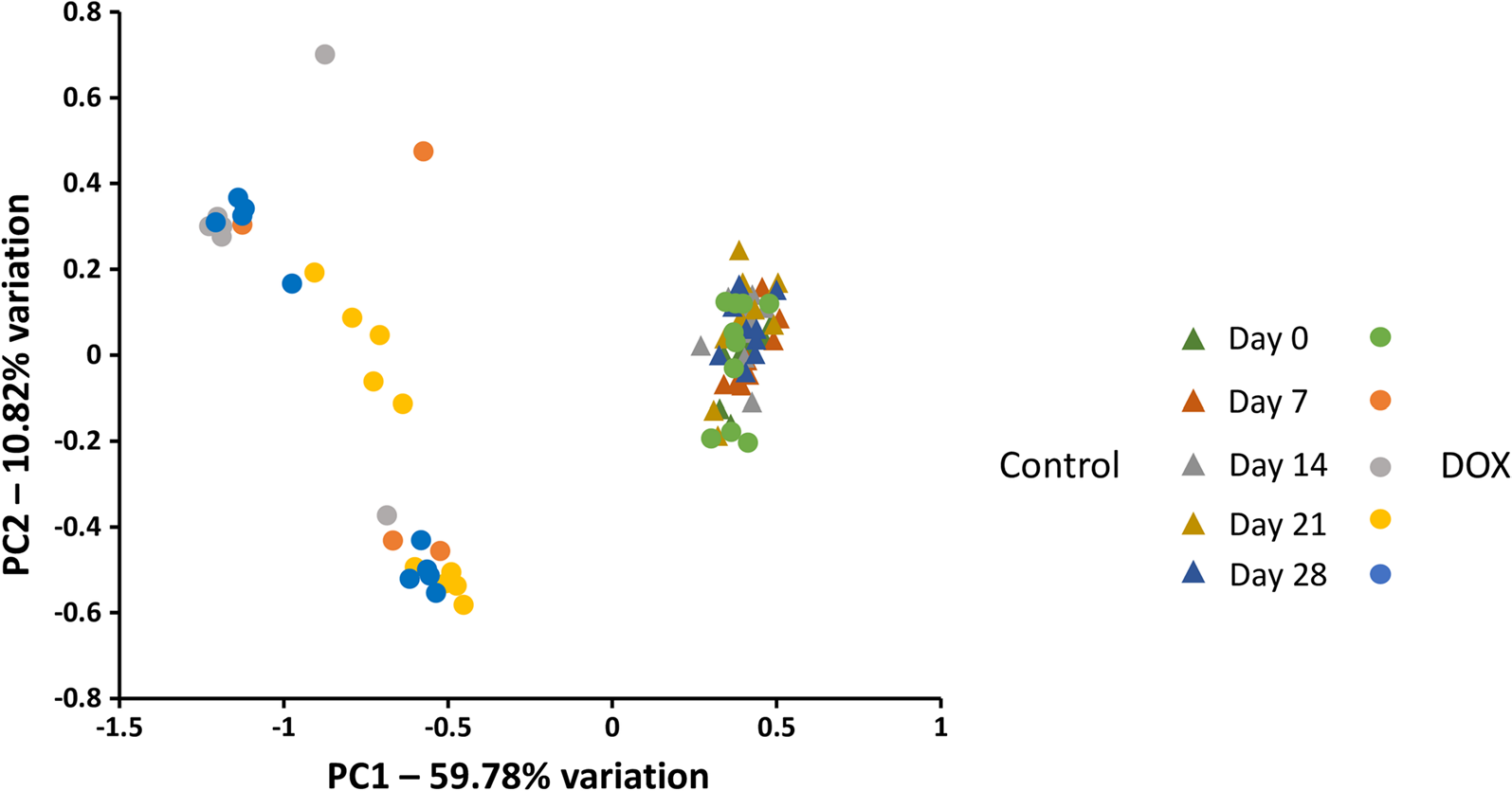
Principal component analysis. Principal component analysis of the fecal microbiota in DOX (circles) and control (triangles) animals. The DOX animals at day 0, and the control animals at all time points cluster tightly

Control animals at all time points and DOX animals at day 0 cluster tightly on principal component analysis (PCA). Post-treatment DOX animals form a looser but distinct cluster (Fig. 3). Testing via two-way PERMANOVA for significant differences in β-diversity confirmed that both treatment and time were significant factors (*p* < 0.0001, F = 95.15 and *p* < 0.0001, F = 8.91, respectively). One-way PERMANOVA, performed to determine pair-wise differences, detected significant differences between the later three time-points of DOX-treated mice and all control time-points (*p* = 0.0135 to 0.0045, F = 47.03 to 208.2). Conversely, within-group differences in DOX-treated mice were limited to differences between the starting time-point and the last three time-points (*p* = 0.0045, F = 59.47 to 103.6). Significant within-group differences in the control group were found between time-points 1 and both 3 (*p* = 0.018, F = 13.44) and 5 (*p* = 0.014, F = 11).

### Discussion

These data indicate that the FM of female C57BL/6NCrl mice is significantly altered by administration of doxycycline at a concentration of 2 mg/ml in the drinking water. This was evidenced by decreased richness and evenness, and disparate populations observed after treatment. They also suggest that after 2 weeks of treatment, effects persist for greater than 1 week, as samples taken at the end of week 3 still exhibited these changes.

The results reported here are not surprising when one considers doxycycline’s spectrum of activity and intestinal route of elimination [42–45]. Though unsurprising, the results are not underwhelming, as they illustrate the occult malleability of animals’ baseline condition. These findings suggest that investigators utilizing tet-on or tet-off models should consider the GM as a variable to be minimized or controlled for in their experiments.

To our knowledge, there are no published recommendations or guidelines to aid investigators, thus we propose use of appropriate controls: ideal control animals are littermates or animals of the same strain and individual history (including source, number of backcrosses if any, sex, age, husbandry management, etc.). Controls must be unresponsive to doxycycline (which requires het X wild type breeding to yield transgene negative animals) and must be given the same dose of doxycycline as experimental animals. Finally, any experimental outcomes that are or may be responsive to changes in the GM must be interpreted with care, especially when comparing the results with other models that do not require doxycycline such as knockouts and Cre/lox models. Alternatively, non-antibiotic analogs of doxycycline could be considered [46, 47].

In summary, the common gene-regulating dose of doxycycline causes a significant dysbiosis in female C57BL/6NCrl mice. This altered microbial community may present challenges or confounds in experimental animals.

## Limitations

### Impact limitation

- Results are/may not be broadly applicable to other study populations e.g. mice of different age, sex, strain, vendor source, diet, disease state, etc.

### Study limitation

- Results do not account for different doses, durations, or routes of administration of doxycycline.
- Results do not include effects of doxycycline on other microbial communities e.g. vaginal, skin etc.
- Study does not control for stochastic influences.

## Additional files

**Additional file 1.** Detailed descriptions of animals and husbandry, welfare assessments and interventions, doxycycline administration, and sample collection that were omitted from the primary manuscript due to length restrictions.

**Additional file 2.** Detailed description of DNA extraction that was omitted from the primary manuscript due to length restrictions.

**Additional file 3.** Fecal microbial community parameter comparisons by treatment and time.This table includes fecal microbial community parameters (mean OTUs per sample, Shannon diversity index, and Chao1) for each sample collected.

**Additional file 4.** Relative abundance at taxonomic level of OTU. Bar charts showing the bacterial composition of the same control and doxycycline-treated mice at all time points annotated to the taxonomic level of OTU. Legend of prominent OTUs is shown below. Each bar represents an individual animal (DOX animals numbered 1–10, control animals numbered 11–20).

## Abbreviations

GM: gut microbiota
FM: fecal microbiota
DOX: doxycycline-treated
OTU: operational taxonomic unit
UC: unclassified
PCA: principal component analysis
