# Supplementary material for: Doxycycline induces dysbiosis in female C57BL/6NCrl mice
Source: BMC Res Notes. 2017 Nov 29;10:644. doi: 10.1186/s13104-017-2960-7 (PMC5708113; doi:10.1186/s13104-017-2960-7)
Supplement: Supplementary file 1 — Additional file 1. Detailed descriptions of animals and husbandry, welfare assessments and interventions, doxycycline administration, and sample collection that were omitted from the primary manuscript due to length restrictions. [file 13104_2017_2960_MOESM1_ESM.docx]

**Animals and Husbandry**

Twenty female C57BL/6NCrl mice were ordered from Charles River Breeding Laboratories and were 8-12 weeks of age at the time of arrival. Animals were housed 5 per cage in individually ventilated cages (Allentown mouse ventilated 75 J) with autoclaved corncob bedding (Bed-O’ Cobs 1/8″ and 1/4″ combination) and cotton and paper nesting material (AnCare Nestlet, Shepherd Specialty Paper Enviro-dri). LabDiet irradiated 5LOD chow was provided ad libitum in wire bar feeders. Water was initially provided via automatic watering system (Lixit®). Three days after arrival, the lixit dispensers were removed and animals were provided distilled water in glass bottles. Room temperature was maintained at 74°F +/- 4°, relative humidity was maintained between 30% and 70%, and automatic timers turned lights on at 6 a.m. and off at 6 p.m. for the duration of the study. Animals were left undisturbed save for the addition of water bottles, for 8 days before the start of the study.

**Welfare Assessments and Interventions**

Animals were observed at least once daily by husbandry staff. Research personnel assessed animals for activity, mentation, and hydration status three times a week. On day 1, 4 of the 5 animals in one DOX cage presented with mild, superficial ulcerations on the dorsal aspects of the tail base. The animal without lesions was presumed to be a potential aggressor, and was removed to a separate cage. Lanolin (Lansinoh) was applied to the tail lesions once daily for 2 days. The lesions were completely healed after 6 days, and the previously separated animal was returned to the home cage. The lesions did not recur, and no inter-animal aggression was observed. On day 7, a DOX animal from the same cage was observed to be mildly dehydrated, hunched and scruffy. 0.5 ml LRS was administered subcutaneously on days 7-9. The dehydration resolved, and the animal remained clinically normal for the duration of the study. No other injuries or adverse consequences were observed.

**Doxycycline Administration**

Doxycycline water was prepared by diluting 800 mg doxycycline hyclate (Sigma 0991-10g) in 400ml Gibco distilled water (ref# 15230-147) to a final concentration of 2 mg/ml. Doxycycline water was stored and administered in amber-colored bottles. Distilled water was provided to control animals throughout the study. Water bottles were replenished, and doxycycline water bottles were agitated by repeated inversion thrice weekly on Mondays, Wednesdays, and Fridays. Fresh water or doxycycline water was provided weekly, and water bottles were changed biweekly.

**Sample Collection**

Fecal pellets were collected from each animal between 9 am and 4 pm in the animal housing room on days 0, 7, 14, 21, and 28. Animals were gently restrained and fresh feces were collected directly into sterile micro centrifuge tubes (Fisherbrand 509-GRDS-FIS) or were collected from the surface of the cage changing station or gloved hand and transferred to tubes using the wooden end of sterile cotton tipped applicators (Medline, MDS202000). Cage changing station surfaces and gloves were sanitized with Spor-Klenz (STERIS) between each animal, and gloves were changed between cages. Only one cage was in the cage changing station at a time, and separate stations were used for the DOX and control animals. DOX animals were always handled before control animals; personnel were not blinded to experimental group. Samples were immediately placed on ice, and were transferred to -80°C freezer within 30 minutes. At the completion of the study, samples were packed on dry ice and shipped overnight to the University of Missouri Metagenomics Center (MUMC) for analysis.
