# Supplementary material for: Doxycycline induces dysbiosis in female C57BL/6NCrl mice
Source: BMC Res Notes. 2017 Nov 29;10:644. doi: 10.1186/s13104-017-2960-7 (PMC5708113; doi:10.1186/s13104-017-2960-7)
Supplement: Supplementary file 2 — Additional file 2. Detailed description of DNA extraction that was omitted from the primary manuscript due to length restrictions. [file 13104_2017_2960_MOESM2_ESM.docx]

**Isopropanol DNA extraction**

Briefly, one fecal pellet was placed into a sterile 2 mL round-bottom tube containing 800 µL lysis buffer (500 mM NaCl, 50 mM Tris-HCl pH 8.0, 50 mM EDTA, and 4% sodium dodecyl sulfate) and a 0.5 cm diameter stainless steel bead. Samples were mechanically disrupted using a TissueLyser II (Qiagen, Venlo, Netherlands) for 3 minutes at 30 Hz, followed by incubation at 70°C for 20 minutes with periodic vortexing. Samples were centrifuged at 5000 × g for 5 min., and the supernatant was then transferred to a sterile 1.5 mL Eppendorf tube containing 200 µL of 10 mM ammonium acetate. Lysates were vortexed, incubated on ice for 5 min., and then centrifuged. Supernatant was transferred to a sterile 1.5 mL Eppendorf tube and one volume of chilled isopropanol was added. Samples were incubated on ice for 30 min. and then centrifuged at 16,000 × g, at 4° C, for 15 min. The resulting DNA pellet was washed with 70% ethanol and resuspended in 150 µL Tris-EDTA (10 mM Tris and 1 mM EDTA), followed by addition of 15 µL of proteinase K and 200 µL of AL Buffer. Samples were incubated at 70°C for 10 min. and 200 µL of 100% ethanol was added to the tubes. Samples were mixed by gentle pipetting and the contents transferred to a spin column from the DNeasy kit. The DNA was purified following the manufacturer’s instructions and eluted in 200 µL EB buffer.
