# Supplementary material for: Doxycycline induces dysbiosis in female C57BL/6NCrl mice
Source: BMC Res Notes. 2017 Nov 29;10:644. doi: 10.1186/s13104-017-2960-7 (PMC5708113; doi:10.1186/s13104-017-2960-7)
Supplement: Supplementary file 3 — Additional file 3. Fecal microbial community parameter comparisons by treatment and time.This table includes fecal microbial community parameters (mean OTUs per sample, Shannon diversity index, and Chao1) for each sample collected. [file 13104_2017_2960_MOESM3_ESM.docx]

|  | **Day** | **Mean OTUs per sample** | **Shannon diversity index** | **Chao1** |
| --- | --- | --- | --- | --- |
| **DOX** | **0** | 45.3 + 2.9 | 4.1 + 0.41 | 621.7 + 28.2 |
|  | **7** | 32.0 + 2.0 | 0.85 + 0.15* | 198.8 + 36.3* |
|  | **14** | 28.4 + 3.6 | 0.69 + 0.09* | 161.6 + 25.1* |
|  | **21** | 31.6 + 3.2 | 1.5 + 0.41* | 204.0 + 54.1* |
|  | **28** | 28.7 + 5.4 | 0.82 + 0.30* | 176.3 + 77.0* |
| **Control** | **0** | 43.3 + 2.9 | 4.2 + 0.14 | 616.4 + 34.3 |
|  | **7** | 41.8 + 1.2 | 4.0 + 0.35 | 643.7 + 28.3 |
|  | **14** | 45.2 + 1.7 | 3.8 + 0.29 | 659.5 + 25.6 |
|  | **21** | 45.7 + 2.0 | 3.7 + 0.39 | 645.8 + 25.9 |
|  | **28** | 45.0 + 2.4 | 3.7 + 0.27 | 648.9 + 34.8 |

* P < 0.001. P-values only reported for statistically significant comparisons between groups at each time point.
