# Supplementary figures and images for: Doxycycline induces dysbiosis in female C57BL/6NCrl mice
Source: BMC Res Notes. 2017 Nov 29;10:644. doi: 10.1186/s13104-017-2960-7 (PMC5708113; doi:10.1186/s13104-017-2960-7)

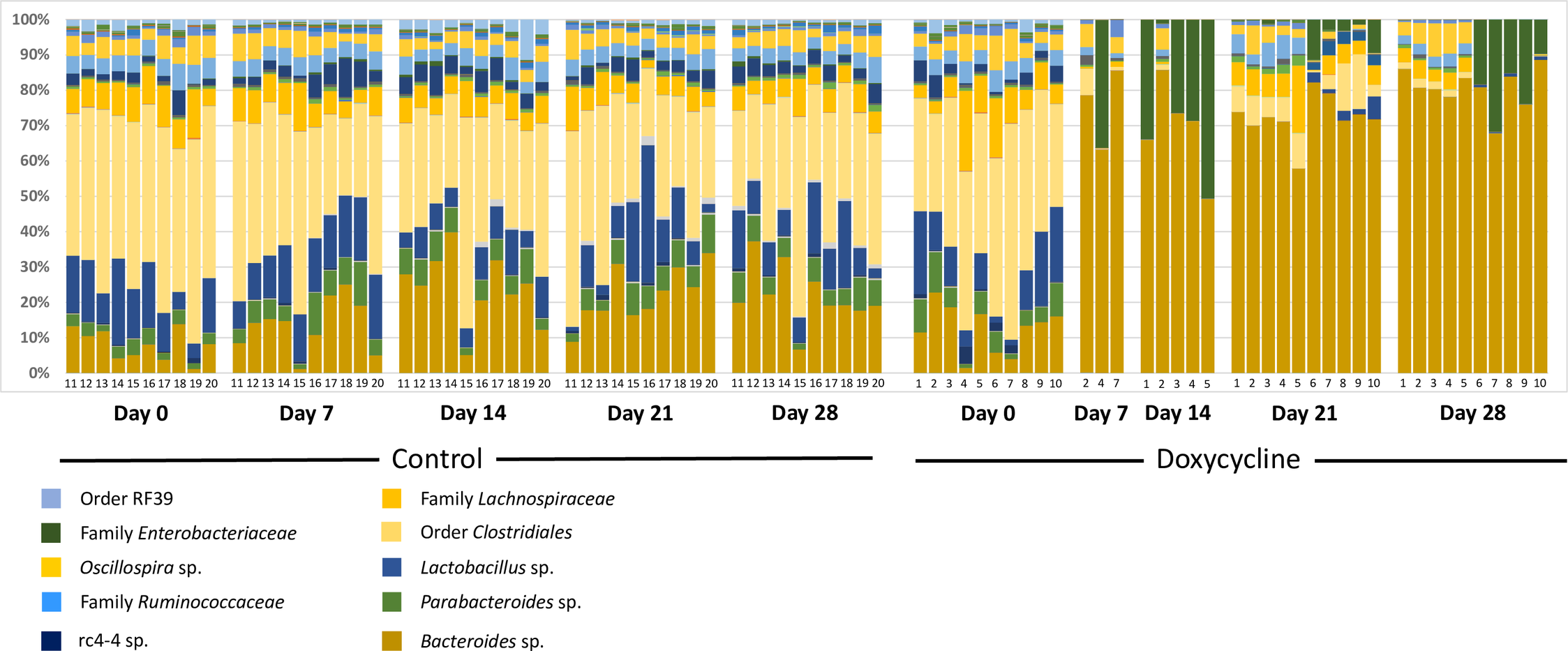

Supplement: Supplementary file 4 — Additional file 4. Relative abundance at taxonomic level of OTU. Bar charts showing the bacterial composition of the same control and doxycycline-treated mice at all time points annotated to the taxonomic level of OTU. Legend of prominent OTUs is shown below. Each bar represents an individual animal (DOX animals numbered 1–10, control animals numbered 11–20). [file 13104_2017_2960_MOESM4_ESM.tif]
